# Effects of different concentrations of *Lactiplantibacillus plantarum* and *Bacillus licheniformis* on silage fermentation parameter, chemical composition and microbial community of *Pennisetum sinese*

**DOI:** 10.3389/fmicb.2025.1532060

**Published:** 2025-03-31

**Authors:** Qiong Chen, Baisheng Yu, Yanchen Zhu, Haoming Xiong, Yongqing Guo, Dewu Liu, Baoli Sun

**Affiliations:** College of Animal Science, South China Agricultural University, Guangzhou, China

**Keywords:** *Pennisetum sinese*, *Lactiplantibacillus plantarum*, *Bacillus licheniformis*, silage, microbial community

## Abstract

The purpose of the experiment was to study the effects of different concentrations of *Lactiplantibacillus plantarum* (LP) and *Bacillus licheniformis* (BL) on the quality of *Pennisetum sinese* (PS) silage. The experiment consisted of seven treatment groups. The control group did not use additives, and the experimental groups were added with LP or BL of 1 × 10^5^ CFU/g fresh weight (FW), 1 × 10^6^ CFU/g FW and 1 × 10^7^ CFU/g FW, respectively. The nutritional value of *Pennisetum sinese* silage was comprehensively evaluated using CNCPS 6.5 system and 16sDNA sequencing technology. The results showed that the ammonia nitrogen content and pH of each experimental group were significantly lower than those of the control group (*p* < 0.05). The starch content gradually decreased and the water-soluble carbohydrate (WSC) content increased in both LP and BL groups with the increase of addition concentration. The LP7 group could significantly increase the true protein content in protein (*p* < 0.05), and CP in BL groups decreased gradually with the increase of concentration. Compared with the control group, the content of neutral detergent fiber (NDF) and acid detergent fiber (ADF) was significantly lower in LP7 group (*p* < 0.05) and the ADF content was significantly lower in BL5 group (*p* < 0.05). In addition, LP and BL were able to change the proportion of each component in CNCPS system for *Pennisetum sinese* silage. The use of LP and BL can reduce the relative abundance of harmful microorganisms in silage such as *Sediminibacterium* and *Nitrospira*, and significantly change the microbial community structure in silage (*p* < 0.05). In conclusion, LP and BL have significant effects on silage quality and nutritional value. The nutritional value of *Pennisetum sinese* in LP groups showed a dose-dependent effect, and adding 1 × 10^7^ CFU/g LP have the best effect in silage. The best effect was achieved by adding 1 × 10^5^ CFU/g BL in BL groups, and the effect of LP7 group was better than that of BL5 group.

## 1 Introduction

With the rapid development of China, Chinese people have gradually incorporated more beef and dairy products into their daily diet, resulting in the sustained and rapid growth of per capita consumption of beef and dairy products, which may drive the further development of ruminant breeding in China (Gao et al., 2023). However, due to the shortage of feed resources and the rising prices of feed, leading to the high production cost of the breeding industry, which is one of the most important factors restricting the development of the modern breeding industry in China (Li et al., 2022). To reduce cost and increase efficiency, we are looking for a new cheap feed resource with high nutritional value.

*Pennisetum sinese* (PS) is a perennial gramineous plant, which was bred by *P. purpureum* and *P. alopecuroides* and widely cultivated in subtropical areas of China. It has the characteristics of strong adaptability, fast growth rate, high yield, good palatability, high crude protein content, more balanced amino acid content, and rich nutrition (Wang et al., 2016). What’s more, it has a very low cost. However, compared with its crude protein, PS has a lower content of crude fiber and water-soluble carbohydrate (WSC), which makes it difficult to obtain high-quality silage through directly ensiling (Li et al., 2018).

According to the former research, silage additives, such as inorganic additives, organic additives, and microbial agents which are usually added in the ensilage process, can effectively improve the nutritional value and feeding value of silage (Muck et al., 2018). Therefore, it’s feasible to improve the quality of PS by adding some kind of silage additives during silage.

*Lactiplantibacillus plantarum* (LP) belongs to heterofermentative lactic acid bacteria (LAB) (Popova-Krumova et al., 2024), which is one of the most commonly used microbial preparations in currently silage production. It also regarded as a safe microbial additives (GRAS micro-organisms), which have been widely used in food preservation. There are studies shown that LP, as biocontrol agents, could be applied to control green mold on citrus fruit (Chen et al., 2021). In addition, many studies have proved that using LP in silage process can inhibit the growth of yeast and mold, which could decrease the nutritional loss caused by aerobic degradation of silage and improve the fermentation quality of silage by producing lactic acid (LA) (Chen et al., 2022; Mu et al., 2021; Yan et al., 2019). The principle of it is probably because it can produce LA to decrease environmental pH (Blajman et al., 2018), inhibit harmful microbial activity, thus reduce the loss of nutrients in feed by reducing the decomposition of protein and WSC (Zhang et al., 2021).

*Bacillus licheniformis* (BL) is a Gram-positive thermophilic bacterium commonly found in soil, which is one of the most exploited groups for biological control of pathogens and pests (Yang et al., 2021). According to the reports of the Panel on Additives and Products or Substances used in Animal Feed (FEEDAP), BL is presumed safe for the environment, the target species and consumers of products from animals fed treated silage (Bampidis et al., 2019). *Bacillus* are known to produce a great variety of metabolites which can inhibit the growth and functions of cellular organisms like bacteria, fungi, insects, nematodes and acellular organisms like viruses (Saxena et al., 2020). Other studies described that *Bacillus* can improved the mean *in vitro* DM and NDF digestibility of different forage sources of varying qualities (based on crude protein content) (Pan et al., 2022). Meanwhile, in our previous experiment on alfalfa silage, we found that silage with LP and BL could significantly improved its *in vitro* fermentation gas production and reduce the ammonia nitrogen content (NH_3_-N) compared with the control group (Zhu et al., 2022). We thus consider that BL has the potential as an efficient silage additive.

Proposed by Cornell University in the 1990s, the Cornell Net Carbohydrate and Protein System (CNCPS) can reflect the nutrient composition and metabolism of ruminant feeds comprehensively. It’s an analytical technique that can combine the conventional nutritional components and degradation characteristics of forage grass and evaluate the nutritional value of forage grass more accurately (Dineen et al., 2021).

Thus, the objectives of this study were to investigate the effect of LP and BL inoculation on fermentation characteristics and chemical composition of PS by CNCPS techniques, and the major microbial groups and bacterial community involved in feed digestion and rumen fermentation, with a view to provide a reference for their application in the silage processing industry.

## 2 Methods

### 2.1 Silage material and preparation

Fresh PS of this study were obtained in a *Pennisetum* planting base in Huaiji County, Zhaoqing City, Guangdong Province, China. The PS was harvested at 2.5–3.0 m by an automatic lawn mower with 20–30 cm height left, and chopped into a size of 2 cm. LP (1.0 × 10^11^ CFU/g) was obtained from College of Forestry and Landscape Architecture, South China Agricultural University and BL (1.0 × 10^10^ CFU/g) was bought from Guangdong Vtr Bio-Tech Co., Ltd. (Zhuhai City, Guangdong Province, China). The experiment consisted of seven treatment groups and ensiled with (1) a control group (CON) with distilled water, (2) 1.0 × 10^5^ CFU/g fresh weight (FW) LP (LP5), (3) 1.0 × 10^6^ CFU/g FW LP (LP6), (4) 1.0 × 10^7^ CFU/g FW LP (LP7), (5) 1.0 × 10^5^ CFU/g FW BL (BL5), (6) 1.0 × 10^6^ CFU/g FW BL (BL6) and (7) 1.0 × 10^7^ CFU/g FW BL (BL7). After mixing with different concentration of LP and BL inoculants respectively, the forage piles were packed into vacuum-sealing polyethylene plastic bags and vacuum-sealed at room temperature (25–28°C) for 35 days. Each of the groups was ensiled using three replicates per group with approximately 1,000 g of forage per bag.

### 2.2 Analyses of the fermentation parameter and chemical composition

Samples of fresh and ensiled PS (5 g) that collected at 35 days were homogenized with 45 mL distilled water in a 50 mL centrifuge tube and stored in a 4°C refrigerator for 24 h. After that, filtered through four layers of medical gauze to collect the filtrate for the determination of fermentation parameters. PH was measured via a glass electrode pH meter (PB-10, Sartorius). LA and volatile fatty acids (VFAs) were tested according to the methods described by Erwin et al. (1961). The concentration of NH_3_-N was quantified using a colorimetric method following Broderick and Kang (1980).

The rest of fresh and ensiled samples was dried at 65°C for 48 h and grounded into powder for the determination of chemical composition. Among that, crude protein (CP), soluble crude protein (SCP) and WSC were tested according to the Association of Official Analytical Chemists (AOAC). The amount of Neutral detergent fiber (NDF) and acid detergent fiber (ADF) were tested according to the method of Van Soest et al. (1991). True protein (TP), non-protein nitrogen (NPN), neutral detergent insoluble crude protein (NDICP), and acid detergent insoluble crude protein (ADICP) were measured according to the procedure of Licitra et al. (1996).

### 2.3 Analyses of the CNCPS fraction

The update version (version 6.5) of CNCPS, according to the described by Higgs et al. (2015), was used to calculate CNCPS carbohydrate and protein fractions. The CNCPS divides carbohydrates into eight fractions, CA1, CA2, CA3, CA4, CB1, CB2, CB3, and CC, and it divides protein into five fractions, PA1, PA2, PB1, PB2, and PC. In current study, CA1 refers sugar, CB1 refers starch, CB2 refers soluble fiber, CB3 refers digestible fiber and CC refers indigestible fiber; PA1 refers ammonia, PA2 refers soluble true protein, PB1 refers insoluble true protein, PB2 refers fiber-bound protein and PC refers indigestible protein.

The calculation of the CNCPS nitrogen fractions were as follows:


$$\begin{array}{c} PA1\left(\%CP\right)=NPN\left(\%SCP\right)\times 0.01\times SCP\left(\%CP\right)PA2\left(\%CP\right)\\ =SCP\left(\%CP\right)-PA1\left(\%CP\right) \end{array}$$

$$\begin{array}{c} \text{PB}1\left(\%CP\right)=100-PA1\left(\%CP\right)-\text{PB}1\left(\%CP\right)\\ -\text{PB}2\left(\%CP\right)-PC\left(\%CP\right) \end{array}$$

$$\text{PB}2\left(\%CP\right)=\text{NDICP}\left(\%CP\right)-\text{ADICP}\left(\%CP\right)$$

$$PC\left(\%CP\right)=\text{ADICP}\left(\%CP\right)$$

The calculation of the CNCPS carbohydrate fractions were as follows:


$$CHO\left(\%DM\right)=100-CP\left(\%DM\right)-EE\left(\%DM\right)-\text{Ash}\left(\%DM\right)$$

$$CA4\left(CHO\%\right)=WSC\left(\%CHO\right)$$

$$CB1\left(CHO\%\right)=\text{Starch}\left(\%CHO\right)$$

$$NFC\left(DM\%\right)=CHO\left(\%DM\right)-NDF\left(\%DM\right)$$

$$\begin{array}{c} CB2\left(CHO\%\right)=NFC\left(CHO\%\right)-CA1\left(CHO\%\right)-CA2\left(CHO\%\right)\\ -CA3\left(CHO\%\right)-CA4\left(CHO\%\right)-CB1\left(CHO\%\right) \end{array}$$

$$CB3\left(CHO\%\right)=NDF\left(\%CHO\right)-CC\left(\%CHO\right)$$

$$CC\left(CHO\%\right)=ADL\left(\%CHO\right)\times 2.4$$

### 2.4 Analyses of bacterial community

Genomic DNA was extracted from silage samples using a DNA kit (D3142, Magen Biotech, Guangzhou, China). The V3–V4 region of 16S rRNA was amplified using specific primers with barcodes. The primer sequences are: 341F (5′-CCTACGGGNGGCWGCAG-3′) and 806R (5′-GGACTACHVGGGTATCTAAT-3′). After purification, the amplified products are linked to sequencing adapters, and sequenced on the Illumina Nova platform. Raw sequencing data were filtered to remove low-quality reads, and paired-end reads were merged into high-quality “Clean Tags.” These tags were clustered into operational taxonomic units (OTUs, groups of similar sequences) using Usearch, and chimeric sequences were removed. Then, based on OTU sequences and abundance data, perform species annotation, species composition analysis, indicator species analysis, α-diversity analysis, *β*-diversity analysis, etc. Among them, α-diversity and β-diversity were analysed, respectively, using the QIIMER (version 1.9.1) and R programme (version 2.5.3) to study the structural differences in microbial communities of samples, and use principal coordinate analysis (PCoA) to display the results. The population-level differences in bacteria between different treatments were analysed by LEfSe analysis (LDA >2, *p* < 0.05).

### 2.5 Statistical analysis

The results of the fermentation parameters, chemical compositions, CNCPS fractions and α-diversity indices were analyzed by using a two-way ANOVA method in the SPSS 25.0 software. The model for data processing was: *Y_ij_* = *μ* + *D_i_* + *A_j_* + (D*A)*_ij_* + *ɛ_ij_*, where *Y_ij_* is the dependent variable, *μ* is the overall mean, *D_i_* is the effect of different concentration of the inoculants, *A_j_* is the effect of different silage inoculants, (D*A)*_ij_* is the interaction effect of different concentration of the inoculants and different silage inoculants, *ɛ_ij_* is the random residual error. The LSD method was adopted for multiple comparisons, and *p* < 0.05 indicated a statistical significance. A Pearson correlation coefficient was adopted for the correlation analysis, with *p* < 0.05 indicating relevance (Hu et al., 2021).

## 3 Results

### 3.1 Fermentation parameters of PS silages

The fermentation parameters affected by LP and BL are shown in Table 1. Concentration significantly affected pH, NH3-N, and LA (*p* < 0.05), and inoculant type influenced AA (*p* < 0.05). Both LP and BL reduced pH and NH3-N during PS silage (*p* < 0.05), with pH decreasing as inoculant concentration increased. Compared with CON, LA content was higher in the silage with LP inoculant, and the variation tendency of LA was the same as LP concentration. Among them, the LA content in LP7 group was significant different from CON (*p* < 0.05). BL also elevated LA but not significantly (*p* > 0.05). On the contrary, the content of AA in the silage with LP inoculant was lower compared with CON, and it decreased with the rise of LP concentration. The content of AA in LP7 was significantly lower than that in CON, whereas BL showed no significant AA reduction (*p* > 0.05), suggesting distinct regulatory roles of LP and BL in LA/AA dynamics. What’s more, PA and BA were undetectable, indicating optimal PS fermentation.

**Table 1:** Fermentation quality of ensiled PS treated with LP and BL.

	Treatments	^1^CON	10^5^ CFU/g	10^6^ CFU/g	10^7^ CFU/g	^3^SEM	*P*		
							^4^A	C	A × C
pH	^1^LP	3.60^a^	3.58^b^	3.56^b^	3.51^c^	0.003	0.717	<0.001	0.588
	^1^BL	3.60^a^	3.57^b^	3.56^b^	3.53^c^				
^2^NH_3_-N %DM	LP	0.89^a^	0.69^b^	0.74^b^	0.73^b^	0.018	0.820	0.012	0.998
	BL	0.89^a^	0.71^b^	0.75^ab^	0.73^b^				
LA %DM	LP	6.90^b^	8.01^b^	8.53^b^	10.61^a^	0.197	0.064	0.001	0.284
	BL	6.90	7.21	8.28	8.53				
AA mmol/L	LP	4.56^a^	3.78^ab^	3.72^ab^	3.13^c^	0.116	0.020	0.145	0.236
	BL	4.56	4.47	4.08	4.49				

^a–c^Means within a column with different superscripts differ (*p* < 0.05).

1 CON, control (no additives); LP, silage inoculant with *Lactiplantibacillus plantarum*; BL, silage inoculant with *Bacillus licheniformis*.

2 DM, dry matter; NH_3_-N, ammonia nitrogen; LA, lactic acid; AA, acetic acid.

3 SEM, standard error of means.

4 A, treatment; C, concentration of inoculant; A × C, the interaction between treatment and concentration of inoculant.

### 3.2 Chemical compositions and CNCPS fraction of PS silage

#### 3.2.1 Chemical compositions of PS silage

Nutritional composition is one of the most critical factors to evaluate the quality of silage. According to the result of the chemical compositions that displayed in Table 2, bacterial inoculant type significantly affected all chemical components except starch and WSC (*p* < 0.05), while concentration impacted all indices except EE (*p* < 0.05). Besides, the interaction of those two factors had a significant effect on the index of DM, WSC and EE (*p* < 0.05). Compared with CON, both LP and BL inoculant increased the content of DM, but the function of the two inoculants is various. In LP groups, only the content of DM in LP5 group was significantly higher than CON (*p* < 0.05), and DM gradually decreased with higher concentration. However, DM in all the groups of BL was significantly higher than CON (*p* < 0.05), and the highest content of DM in BL7 group was significantly higher than BL6 and BL7 (*p* < 0.05), which was different with the LP groups. The results of OM and Ash showed that both LP and BL inoculants could decrease the loss of organic matter during PS silage. While LP groups had no significant difference with CON (*p* > 0.05), and the content of OM decreased with LP concentration increased. Unlike the LP groups, OM in BL5 and BL7 was significantly higher than CON (*p* < 0.05). For WSC, LP groups was lower than CON, with LP5 as the lowest and it was significantly lower than other groups (*p* < 0.05). But with the ascend of LP concentration, the content of WSC ascend gradually as well. Similarly, BL groups exhibited rising WSC with concentration, and WSC in BL7 was significantly higher than CON (*p* < 0.05). When the concentration of LP and BL inoculant reached 10^5^ CFU/g respectively, the content of starch in LP and BL groups was significantly higher than CON (*p* < 0.05), which was 12.47 and 4.62% higher than CON, respectively. While with rising concentration, both LP and BL groups had a decrease in starch, among them, the content of starch in LP7 and BL7 was lower than CON with the value of 11.78 and 9.93%, respectively.

**Table 2:** Chemical composition of ensiled PS treated with LP and BL.

	Treatments	CON	10^5^ CFU/g	10^6^ CFU/g	10^7^ CFU/g	SEM	*p*		
							A	C	A × C
DM	LP	19.28^b^	23.50^aA^	19.42^bB^	19.29^bB^	0.053	<0.001	<0.001	<0.001
	BL	19.28^c^	21.60^bB^	21.37^bA^	22.20^aA^				
OM	LP	90.69	91.29	91.24	91.00^B^	0.066	0.035	0.003	0.072
	BL	90.69^c^	91.54^ab^	91.26^bc^	91.95^aA^				
Ash	LP	9.31	8.71	8.76	9.00^A^	0.066	0.035	0.003	0.072
	BL	9.31^a^	8.46^bc^	8.74^ab^	8.49^cB^				
WSC	LP	1.43^a^	0.80^cB^	1.26^bB^	1.31^abB^	0.017	<0.001	<0.001	<0.001
	BL	1.43^ab^	1.33^bA^	1.44^abA^	1.56^aA^				
Starch	LP	4.33^ab^	4.87^a^	4.08^b^	3.82^b^	0.087	0.779	0.024	0.815
	BL	4.33	4.53	4.15	3.90				
EE	LP	2.07^b^	1.87^bB^	2.2^b^	2.67^aA^	0.042	0.700	0.140	0.001
	BL	2.07^b^	2.6^aA^	2.2^ab^	2.07^bB^				

DM, dry matter; OM, organic matter; WSC, water soluble carbohydrate; EE, ether extract. ^A–C^Means within a row with different superscripts differ (*p* < 0.05). ^a–c^Means within a column with different superscripts differ (*p* < 0.05).

#### 3.2.2 Nitrogen fractions of PS silage

Table 3 showed that the effect of different inoculants and concentration to nitrogen fraction of PS. According to the Nitrogen fraction, inoculant type significantly affected CP and ADICP (*p* < 0.05), and concentration influenced all nitrogen indices except NDICP and ADICP (*p* < 0.05), while the interaction of those two factors had a significant effect on all index except SCP (*p* < 0.05). CP content in LP6 and LP7 had no significant difference with CON (*p* > 0.05). Instead, BL6 and BL7, respectively, reduced CP by 9.22 and 15.32% compared with CON (*p* < 0.05). Similarly, both LP and BL groups had lower SCP and NPN than CON, but higher TP content. ADICP content in all LP groups was lower than CON, and LP7 group was the lowest one. On the contrary, the content of ADICP in all BL groups was higher than CON, in which BL7 was the highest one, and BL6 and BL7 was significantly higher than LP6 and LP7 (*p* < 0.05).

**Table 3:** Nitrogen fractions of ensiled PS treated with LP and BL.

	Treatments	CON	10^5^ CFU/g	10^6^ CFU/g	10^7^ CFU/g	SEM	*p*		
							A	C	A × C
CP (%DM)	LP	8.68^a^	7.47^b^	8.67^a^	8.61^aA^	0.074	0.020	0.002	0.003
	BL	8.68^a^	8.00^ab^	7.88^b^	7.35^bB^				
SCP	LP	63.81^a^	58.88^b^	61.91^ab^	58.49^bB^	0.442	0.061	0.040	0.107
	BL	63.81	61.98	60.97	62.93^A^				
NPN	LP	61.34^a^	55.09^cB^	59.53^abA^	57.67^b^	0.377	0.179	0.017	0.009
	BL	61.34^a^	60.67^abA^	56.74^bB^	59.11^ab^				
TP	LP	38.66^c^	44.91^aA^	40.47^bcB^	42.33^b^	0.454	0.179	0.017	0.009
	BL	38.66^b^	39.33^abB^	43.26^aA^	40.89^ab^				
NDICP	LP	20.29^b^	23.13^aA^	20.00^b^	20.16^b^	0.274	0.424	0.532	0.025
	BL	20.29	19.45^B^	20.58	21.47				
ADICP	LP	4.36	4.27	4.22^B^	3.49^B^	0.175	<0.001	0.174	0.009
	BL	4.36^b^	6.24^ab^	5.72^abA^	7.44^aA^				
PA1	LP	61.34^a^	55.09^cB^	59.53^ab^	57.67^b^	0.377	0.179	0.017	0.009
	BL	61.34^a^	60.67^abA^	56.74^b^	59.11^ab^				
PA2	LP	2.47	3.79	2.38^B^	0.83^B^	0.258	0.268	0.554	0.010
	BL	2.47^b^	1.32^c^	4.23^aA^	3.82^aA^				
PB1	LP	15.89^c^	17.99^b^	18.08^b^	21.34^aA^	0.323	0.081	0.037	0.008
	BL	15.89	18.56	18.45	15.60^B^				
PB2	LP	15.94^b^	18.86^aA^	15.78^b^	16.67^ab^	0.301	0.001	0.756	0.022
	BL	15.94	13.21^B^	14.86	14.02				
PC	LP	4.36	4.27	4.22^B^	3.49^B^	0.175	<0.001	0.174	0.009
	BL	4.36^b^	6.24^ab^	5.72^abA^	7.44^aA^				

CP, crude protein; SCP, soluble crude protein; NPN, non-protein nitrogen; TP, true protein; NDICP, neutral detergent insoluble crude protein; ADICP, acid detergent insoluble crude protein; PA1, instantaneously soluble protein or non-protein nitrogen; PB1, rapid degradable true protein; PB2, intermediately degradable true protein; PC, undegradable true protein or acid detergent insoluble crude protein. ^A–C^Means within a row with different superscripts differ (*p* < 0.05). ^a–c^Means within a column with different superscripts differ (*p* < 0.05).

From the nitrogen fraction of CNCPS, we could know that inoculant type significantly affected PB2 and PC (*p* < 0.05), and concentration had a significant effect on PA1 and PB2 (*p* < 0.05), the interaction of those two factors significantly influenced nitrogen fraction as well (*p* < 0.05). Both LP and BL inoculant could reduce PA1. In LP groups, PA2 decreased with the increase of LP concentration, and among them, LP7 was significantly lower than CON (*p* < 0.05). PB1 in LP groups was significantly higher than CON (*p* < 0.05), and it showed the same trend with concentration. However, BL groups showed an inverse trend and had no significant difference with CON (*p* > 0.05). PB2 increased with the application of LP inoculant but decreased with BL. PC in LP groups was non-significantly lower than CON (*p* > 0.05), while the content of PC in BL groups was higher than CON, and BL7 reached a significant level compared with CON (*p* < 0.05).

#### 3.2.3 Carbohydrate fractions of PS silage

As we could known from Table 4, inoculant type significantly affected ADF, HC, and CHO (*p* < 0.05), concentration impacted all indices except ADL (*p* < 0.05), while the interaction of those two factors influenced all carbohydrate indices except ADL and NFC (*p* < 0.05). NDF and ADF in LP groups was lower than CON, among them the content of NDF, ADF, HC and CHO in LP7 was lowest, which was significantly lower than LP5 (*p* < 0.05). Compared with CON, ADF in BL5 and BL6 was significantly decrease (*p* < 0.05), and HC, ADL, NFC increased significantly (*p* < 0.05).

**Table 4:** Carbohydrate fractions of ensiled PS treated with LP and BL.

	Treatments	CON	10^5^ CFU/g	10^6^ CFU/g	10^7^ CFU/g	SEM	*p*		
							A	C	A × C
NDF	LP	58.25^b^	59.84^aA^	56.25^c^	56.19^c^	0.203	0.441	0.047	0.003
	BL	58.25	57.30^B^	57.75	58.50				
ADF	LP	36.76^a^	36.86^aA^	35.23^b^	35.20^b^	0.140	0.032	0.002	0.031
	BL	36.76^a^	34.65^bB^	34.58^b^	35.42^ab^				
HC	LP	21.48^b^	22.98^a^	21.02^bB^	20.99^bB^	0.099	<0.001	0.002	<0.001
	BL	21.48^b^	22.65^a^	23.17^aA^	23.08^aA^				
ADL	LP	3.25	4.50	5.22	4.60	0.209	0.922	0.051	0.768
	BL	3.25^b^	4.72^a^	4.44^ab^	4.99^a^				
CHO	LP	79.94^b^	81.96^aA^	80.37^b^	79.72^bB^	0.148	0.044	0.014	0.002
	BL	79.94^b^	80.94^abB^	81.18^ab^	82.53^aA^				
NFC	LP	21.69^c^	22.12^bcB^	24.12^a^	23.54^ab^	0.195	0.405	0.004	0.271
	BL	21.69^b^	23.64^abA^	23.43^ab^	24.04^a^				
CA4	LP	1.79^a^	0.98^cB^	1.56^bB^	1.64^ab^	0.019	<0.001	<0.001	<0.001
	BL	1.79^ab^	1.64^bA^	1.77^abA^	1.89^a^				
CB1	LP	5.42^ab^	5.94^a^	5.08^b^	4.79^c^	0.112	0.677	0.033	0.932
	BL	5.42	5.60	5.10	4.73				
CB2	LP	19.92^b^	20.07^b^	23.37^a^	23.09^a^	0.287	0.974	0.007	0.260
	BL	19.92	21.96	22.00	22.50				
CB3	LP	63.13^a^	59.83^ab^	54.41^bB^	56.65^ab^	0.587	0.950	0.002	0.294
	BL	63.13^a^	56.79^b^	58.02^bA^	56.37^b^				
CC	LP	9.74	13.19	15.57	13.82	0.618	0.848	0.057	0.768
	BL	9.74^b^	14.00^a^	13.11^ab^	14.51^a^				

NDF, neutral detergent fiber; ADF, acidic detergent fiber; HC, hemicellulose; ADL, acid detergent lignin; CHO, carbohydrate; NFC, non-fiber carbohydrate; CA, carbohydrate fractionations, i.e., sugars; CB1, starch; CB2, soluble fiber; CB3, available neutral detergent fiber-NDF; CC, unavailable neutral detergent fiber.. ^A–C^Means within a row with different superscripts differ (*p* < 0.05). ^a–c^Means within a column with different superscripts differ (*p* < 0.05).

From the carbohydrate fractions of CNCPS, we could know that both inoculant and the interaction of inoculant and concentration significantly affected CA4 (*p* < 0.05), and concentration had a significant impact on all carbohydrate indices except CC (*p* < 0.05). Compared with CON, LP and BL inoculant could both increased CB2 and CC but decreased CB3. In addition, CA4 in LP and BL groups increased with the rise of concentration, while CB1 showed an inverse trend. Among them, CB1 in both LP5 and LP7 group reached a significant level (*p* < 0.05), but it had no significant difference in BL groups (*p* > 0.05).

### 3.3 Bacterial community in PS silage

#### 3.3.1 Bacterial diversity

Alpha-diversity reflect both the number of species (richness) and their abundance distribution (evenness), to assess treatment or environmental impacts on community structure. From Table 5, we could know that the application of LP and BL inoculant had a great impact on bacterial community of α-diversity. The index of Goods refers to the coverage of all kinds of text libraries, the higher the value, the lower the probability that the sequence in the sample is not detected. In this study, the index of Goods in every groups exceeded 0.99, which indicated that the sequencing coverage was comprehensive and the sequencing results were reliable. The factor of concentration significantly affected α-diversity (*p* < 0.05), inoculant and the interaction of concentration and inoculant significantly influenced Ace and Chao1 index as well (*p* < 0.05). Furthermore, Ace in LP7 was significantly lower than LP6 (*p* < 0.05), but it had no significant difference with CON (*p* > 0.05). Similarly, Ace in BL groups was significantly lower than CON (*p* < 0.05), but it had no significant difference in various concentration (*p* > 0.05). Chao1 index in both LP and BL groups had no significant impact with CON (*p* > 0.05), while LP groups were bigger than BL groups when the concentration was 10^5^CFU/g and 10^6^CFU/g (*p* < 0.05). In addition, Shannon index in LP groups gradually descended when the concentration grew up, among them, LP7 was significantly lower than CON and LP5 (*p* < 0.05), while it had no significant difference between BL groups and CON. Simpson index in LP5 and BL groups was significantly higher than CON (*p* < 0.05).

**Table 5:** Effects of different concentrations of LP and BL on α-diversity in PS silage.

	Treatments	CON	10^5^ CFU/g	10^6^ CFU/g	10^7^ CFU/g	SEM	*P*		
							A	C	A × C
Ace	LP	727.97^ab^	781.09^ab^	815.83^aA^	500.97^b^	23.930	<0.001	0.003	0.004
	BL	727.97^a^	334.18^b^	294.91^bB^	337.73^b^				
Chao1	LP	689.26	737.17^A^	767.64^A^	489.68	22.173	<0.001	0.005	0.006
	BL	689.26	336.81^B^	301.48^B^	343.67				
Shannon	LP	2.98^a^	3.06^aA^	2.65^ab^	2.49^bB^	0.051	0.611	0.035	0.360
	BL	2.98	2.69^B^	2.68	2.61^A^				
Simpson	LP	0.64^b^	0.75^aA^	0.68^b^	0.66^b^	0.005	0.560	<0.001	0.077
	BL	0.64^b^	0.72^aB^	0.70^a^	0.70^a^				
Goods	LP	0.9975	0.9989	0.9987	0.9993	<0.001	0.353	0.039	0.879
	BL	0.9975	0.9996	0.9997	0.9995				

^A–C^Means within a row with different superscripts differ (*p* < 0.05). ^a–c^Means within a column with different superscripts differ (*p* < 0.05).

**Figure 1:**
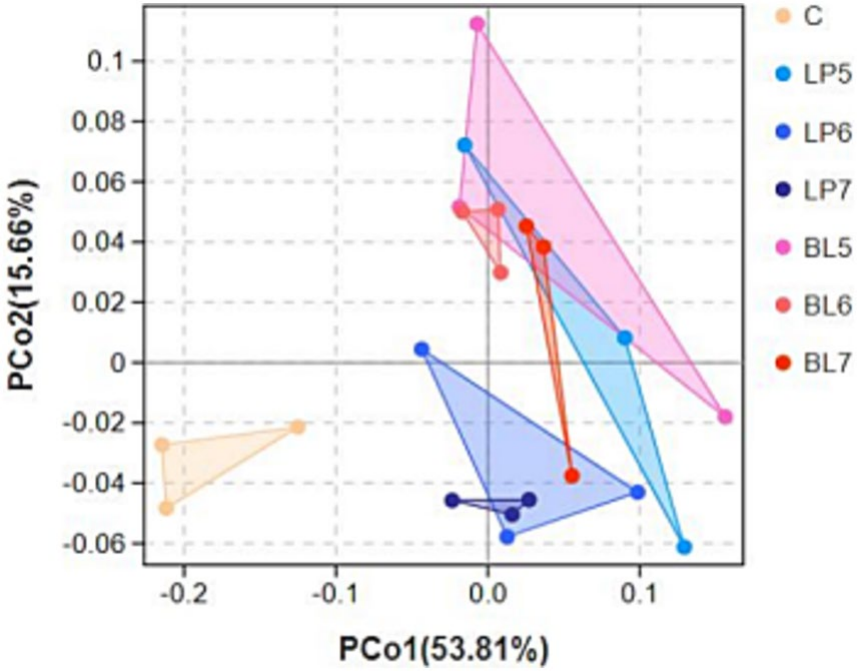
Principal coordinate analysis (PCoA) showing diversities of the bacterial communities with different treatments in PS silage (C, CON; LP, *Lactiplantibacillus plantarum*; BL, *Bacillus licheniformis*).

Principal coordinate analysis (PCoA) was performed for β-diversity which was shown in Figure 1. The chart showed that each group treated with bacterial inoculants had a remarkable separation with CON, which indicated that bacterial inoculant could influence bacterial diversity in PS silage. Furthermore, as can be seen from the chart that LP5 and BL5 had not been completely separated, but with concentration increasing, LP and BL groups gradually distinguished until LP7 and BL7 completely separated. Among LP groups, LP6 is obviously distinguished with LP7, and BL6, BL5 and BL7 had a phenomenon of separation as well, which indicated that LP and BL inoculant could significantly effect bacterial community in PS silage. However, the function of those two inoculants to the bacterial community might be various.

#### 3.3.2 Bacterial composition

**Figure 2:**
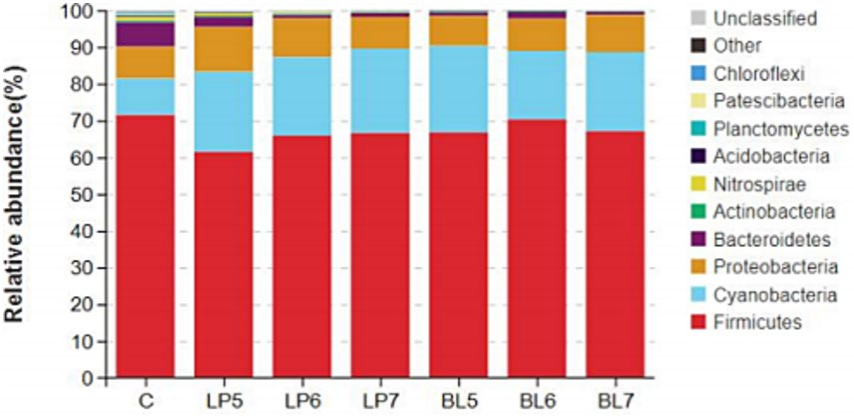
Accumulation map of bacterial communities at the phylum levels for PS silage (C, CON; LP, *Lactiplantibacillus plantarum*; BL, *Bacillus licheniformis*).

**Figure 3:**
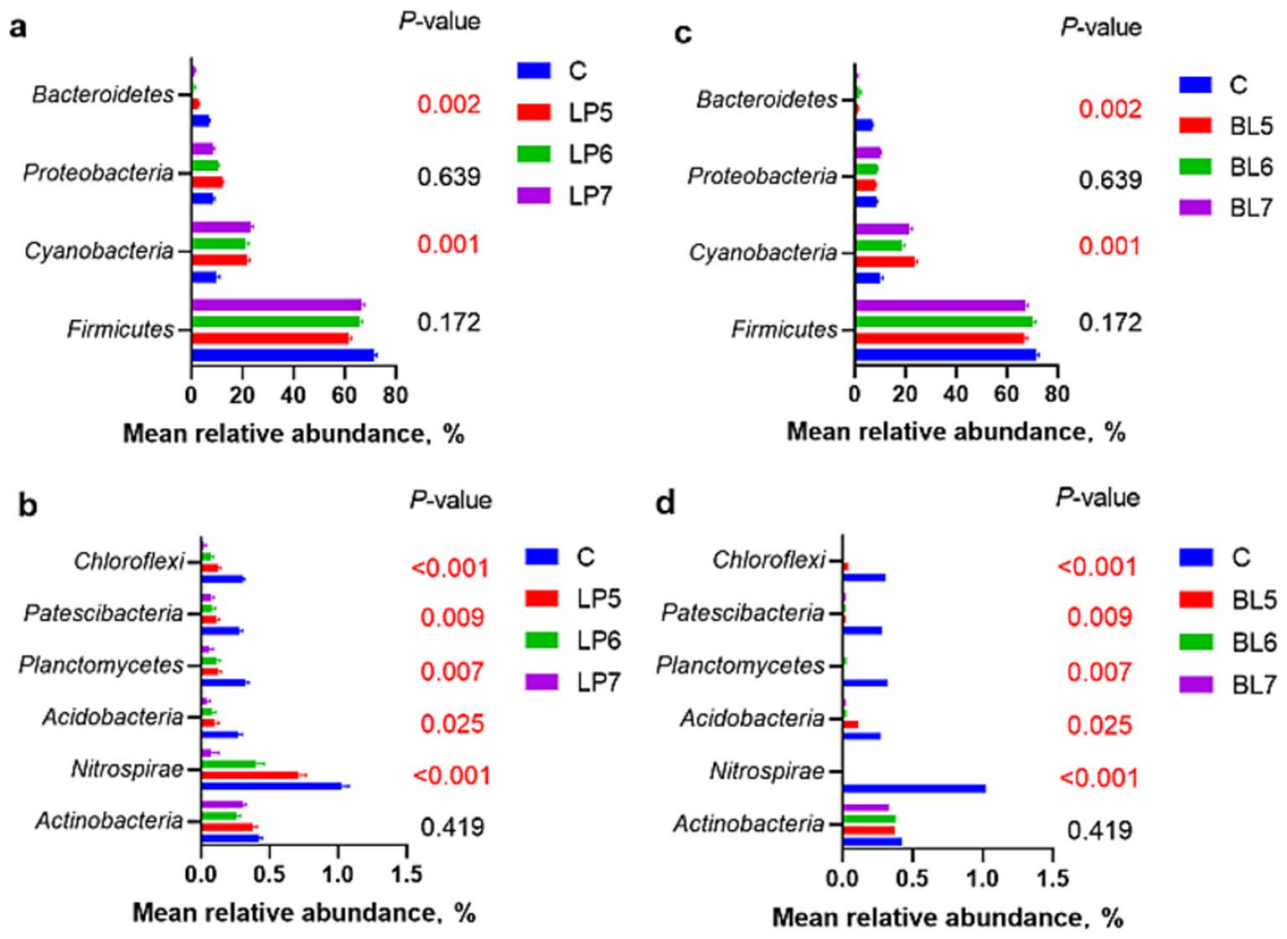
Effect of LP inoculant **(a,b)** and BL inoculant **(c,d)** in silage of PS on mean relative abundance of silage bacteria at phylum level that accounted for ≥1% (C, CON; LP, *Lactiplantibacillus plantarum*; BL, *Bacillus licheniformis*).

From the level of phylum (Figures 2, 3), the dominant bacterial phyla in each group of the experiment were *Firmicutes*, *Cyanobacteria*, *Proteobacteria*, and *Bacteroidetes*. Compared with CON, the relative abundance of *Firmicutes* showed no significant change in all experimental groups except LP5. Both LP and BL increased the relative abundance of *Cyanobacteria* and decreased that of *Bacteroidetes*, but had opposite trends on the relative abundance of *Proteobacteria*. In LP groups, the relative abundance of *Firmicutes* increased with the rising LP concentration, while that of *Proteobacteria* gradually decreased. In BL groups, the relative abundance of *Proteobacteria* gradually increased with the rising concentration. It can also be seen from the Figure 2 that the relative abundance of each phylum in LP7 and BL5 was basically the same. In addition, as the concentrations of LP and BL increased, the relative abundance of *Nitrospirae*, *Acidobacteria* and *Chloroflexi* all gradually decreased, and were significantly lower than that of CON at the concentration of 10^7^ CFU/g FW (*p* < 0.05).

**Figure 4:**
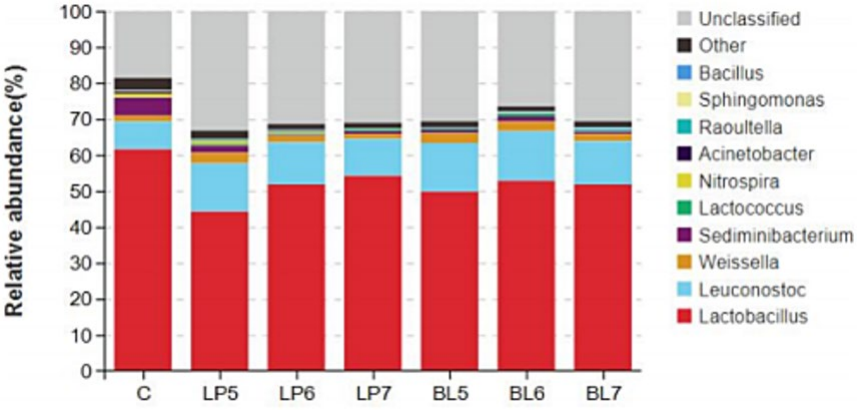
Accumulation map of bacterial communities at the genus levels for PS silage (C, CON; LP, *Lactiplantibacillus plantarum*; BL, *Bacillus licheniformis*).

**Figure 5:**
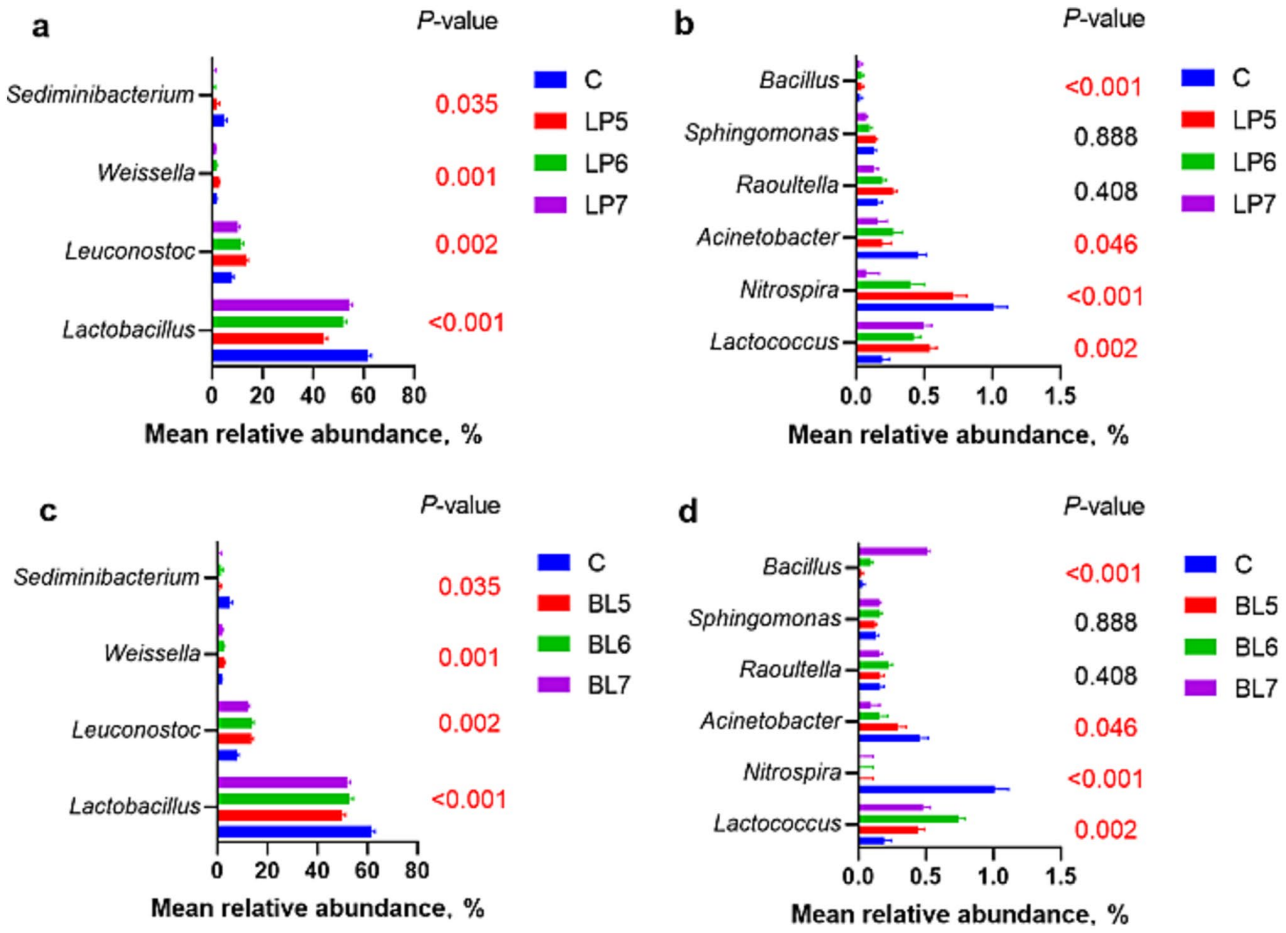
Effect of LP inoculant **(a,c)** and BL inoculant **(b,d)** in silage of PS on mean relative abundance of silage bacteria at genus level that accounted for ≥1% (C, CON; LP, *Lactiplantibacillus plantarum*; BL, *Bacillus licheniformis*).

At the genera level (Figures 4, 5), the dominant bacterial genera in each group were *Lactobacillus*, *Leuconostoc*, *Weissella*, and *Sediminibacterium*. Compared with CON, the relative abundance of *Lactobacillus* decreased (*p* < 0.05) in all treatment groups, but *Leuconostoc* and *Lactococcus* significantly increased, with significant differences from CON at 10^5^ CFU/g FW and 10^6^ CFU/g FW (*p* < 0.05). Among the genera with relative abundance greater than 1%, as LP concentration increased, the relative abundance of *Lactobacillus* continued to rise, while *Leuconostoc*, *Weissella*, *Nitrospira*, *Raoultella*, *Sphingomonas*, and *Bacillus* gradually decreased. In BL groups, the relative abundance of *Lactobacillus* and *Leuconostoc* both first increased and then decreased with BL concentration increasing. *Weissella*, *Nitrospira*, and *Acinetobacter* all decreased with the rising BL concentration. In addition, *Weissella* was higher but *Nitrospira* and *Acidobacter* were lower than that of CON. And *Bacillus* increased with the rising BL concentration. LP and BL could change the composition of the microbial community in PS silage, and different effects would be produced as the concentration varies.

#### 3.3.3 Bacterial LEfSe analysis

**Figure 6:**
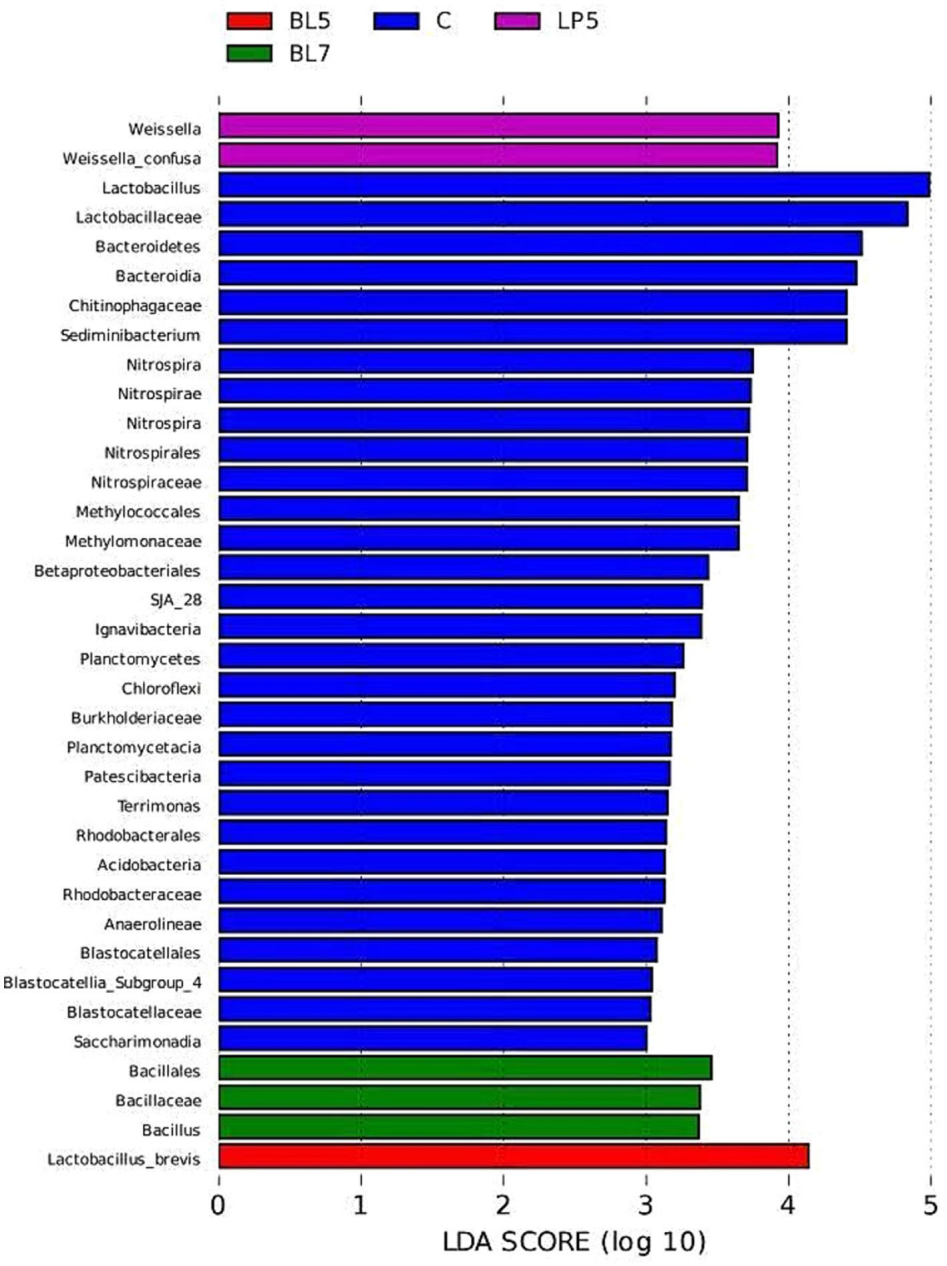
LDA bar chart of silage microorganisms in PS silage.

**Figure 7:**
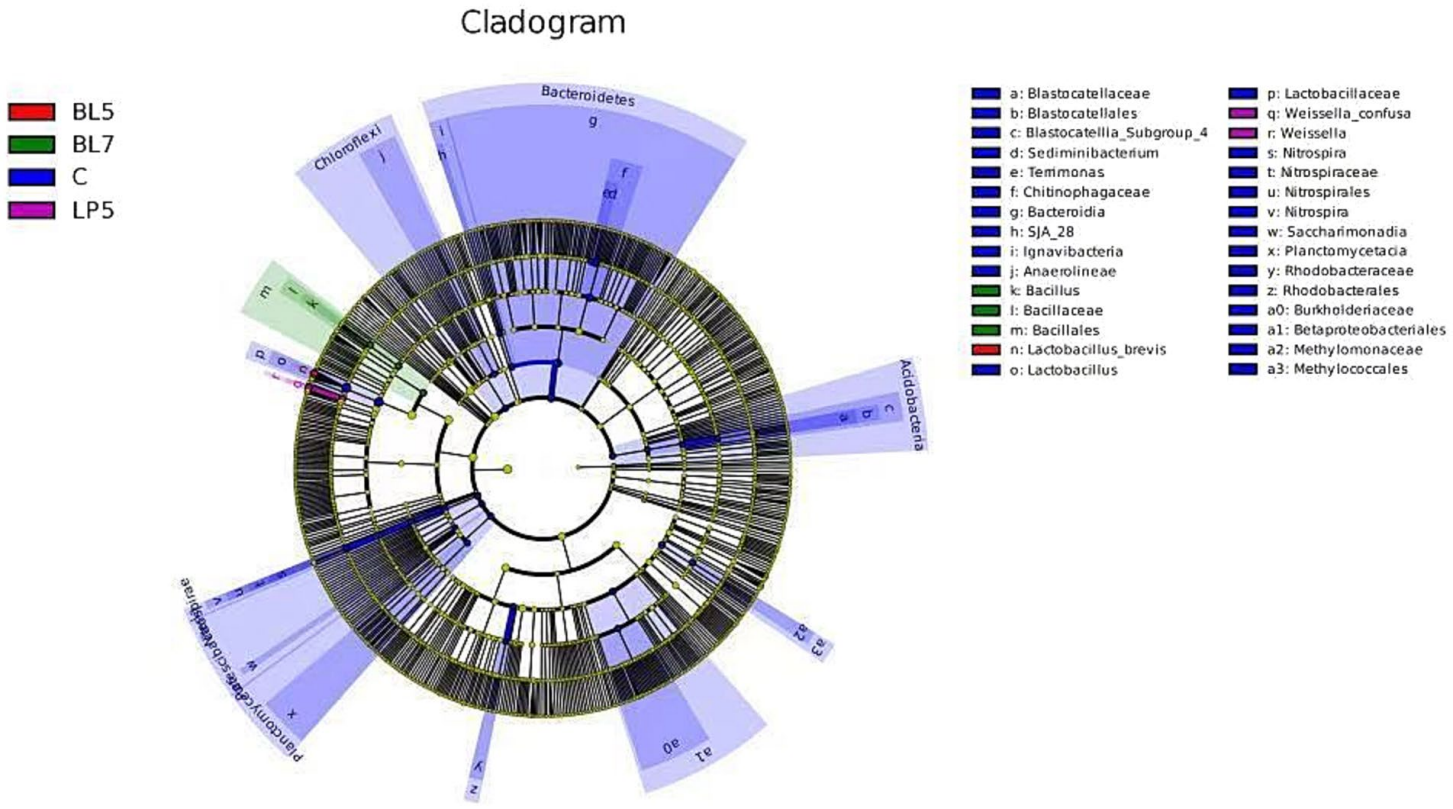
Evolutionary branch diagram of silage microorganisms in PS silage.

To study the differences in microorganisms among different groups, this experiment screened 36 differential bacteria through LEfSe analysis (LDA >2, *p* < 0.05), as shown in Figure 6. These included six phyla, seven classes, seven orders, eight families, six genera, and two species, mainly present in four treatment groups, among which the control group contains 30 differential bacteria. The differential bacterial genera in each treatment group were *Lactobacillus*, *Sediminibacterium*, *Weissella*, *Nitrospira*, *Bacillus*, and *Terrimonas*. From Figure 7, it could be seen that there were multiple differential evolutionary branches in CON, while the differential bacteria in the treatment groups were all on the same evolutionary branch.

## 4 Discussion

pH is one of the important indicators to evaluate the quality of silage. Acidic environments can inhibit the activity of harmful microorganisms during the silage process, so the pH below 4.2 is considered one of the criteria for high-quality silage (Zi et al., 2021). In this experiment, the pH of each treatment group was between 3.51 and 3.6, indicating sufficient fermentation, in line with the standards of high-quality silage. With the increase of additives, the pH gradually decreases, which is consistent with the research result of Li et al. (2018), that *Lactiplantibacillus plantarum* can reduce the pH of PS silage. However, when the silage pH is below 3.5, excessive acidification may occur, leading to a decline in feed quality, affecting the production performance of animals, so it should be used in conjunction with other feeds during practical application. NH_3_-N is an index reflecting the decomposition degree of amino acids and proteins in silage. The higher the value is, the greater the decomposition degree of protein is, and the worse the fermentation quality is Kung et al. (2018). Moreover, the capacity of NH_3_-N to be absorbed and utilized in animals is far lower than that of true protein, so the higher the content of NH_3_-N, the lower nutritional value of feed. The NH_3_-N content of the experimental groups was significantly lower than that of CON, which may be because LP and BL can inhibit the activity of harmful microorganisms such as mold and clostridium in silage, effectively avoiding the complete degradation of feed protein into NH_3_-N, and improving the feed nutritional value (Ji et al., 2020). LP is a homofermentative LAB that can promote the production of LA, and LA content shows a dose-dependent effect with additive concentration, which is consistent with the results of this experiment (Liu et al., 2016). The LA content in BL groups gradually increased with the rise of BL addition concentration. This may be because BL is an aerobic microorganism with a unique biological oxygen-grabbing mechanism, which can rapidly consume the oxygen in silage, create anaerobic conditions, inhibit the growth of miscellaneous bacteria, promote the proliferation of LAB to produce more LA. As a homofermentative LAB, LP only produces LA as a metabolic product when decomposing glucose, while heterofermentative LAB also produce AA, ethanol, CO_2_ and other products (Oliveira et al., 2017). The decrease of AA content with the increase of additive concentration in LP groups indicates that there are heterofermentative LAB producing AA in the original microbial community of PS. And as the addition of LP increases, homofermentative LAB gradually take the dominant position, then the content of AA gradually decreases. There was no significant difference in AA content between CON and BL groups, which may be because BL has a small impact on the composition of LAB in silage.

As plant respiration and the intense activity of aerobic microbes during the silage process, nutrients are constantly consumed, producing water, CO_2_ and free ammonia, which leads to the decrease of DM content in silage (Ridwan et al., 2015). As a homofermentative LAB, LP can quickly produce a large amount of LA during storage, reducing the environmental pH, inhibiting the activity of harmful microorganisms such as yeast and mold, avoiding nutrient loss caused by aerobic degradation of silage, and significantly improving the success rate and nutritional value of silage (Chen et al., 2022). This was consistent with the results of this study, and DM content in LP groups was higher than that in CON. However, as the addition of LP increased, the consumption of nutrients by the exogenous bacteria also increased, and then produced substances such as water and CO_2_, leading to a gradual decrease in DM content in LP6 and LP7. BL can produce active substances with antibacterial effects and has a unique biological oxygen-grabbing mechanism, which can inhibit the growth and reproduction of pathogens and aerobic microorganisms (Chen et al., 2022) and reduce the consumption of nutrients (Pattnaik et al., 2001). This may be the reason for the higher DM content in BL groups compared to CON. The results of OM can also lead to the same conclusion. The OM content in all treatment groups is higher than that in the control group. And the OM content in LP groups decreases with the increase of concentration, while the OM content in BL groups shows the same upward trend as the addition concentration. WSC is an energy source for microbial activity in silage, and the vigorous activity of microorganisms is usually accompanied by rapid consumption of WSC (Amer et al., 2012). When LP is added to the silage, the WSC content in LP5 is significantly lower than that in CON due to the consumption of WSC with the growth activity of LP. As the LP content increases, the WSC content gradually rises. On the one hand, this is because high concentration of LP inhibits the consumption of WSC by harmful microorganisms. On the other hand, research has shown that LP can promote the degradation of fiber or macromolecular carbohydrates in silage to produce small molecular carbohydrates, thereby increasing the WSC content. The results of starch in this experiment also prove this point. The amylase produced by *Lactiplantibacillus plantarum* can break down starch in silage into reducing sugars such as glucose and lactose (Li and Liu, 2021). At low concentration, the WSC content in the feed is sufficient to meet the needs of microbial activity in the silage. Therefore, the starch content in LP5 is significantly higher than that in CON. As the concentration of LP increases, the ability of *Lactiplantibacillus plantarum* to break down complex carbohydrates such as starch strengthens, leading to a gradual decrease in Starch content and an increase in WSC content. BL is also the case, as it can not only inhibit the consumption of WSC in silage by harmful microorganisms, but its strong cellulase and amylase activities can promote the degradation of fiber and starch to produce WSC. Moreover, BL may reduce its consumption of WSC by adjusting metabolic pathways under anaerobic conditions. For instance, in corn silage fermentation, the addition of BL has not been observed to significantly decrease WSC content (Yin et al., 2024), which may be related to its metabolic characteristics of preferentially utilizing other substrates or reducing the breakdown of nutrients under oxygen-limited conditions. Therefore, the WSC content in BL groups is significantly higher than that in LP groups at the same concentration. The starch content in BL groups is lower than that in LP groups at the same concentration, possibly because BL has higher amylase activity and is more efficient in breaking down starch. A large number of studies have shown that the addition of LP can significantly increase the EE content in silage with a dose-dependent effect, which is consistent with this experiment. In contrast, BL has higher lipase activity, so the EE content in BL groups gradually decreases with the increase of BL concentration.

CP is one of the most important nutrient elements in feed, the content of CP is an important indicator of evaluating the quality of silage. In this experiment, the protein content of LP5 was lower than that of CON, possibly because the concentration of LP was too low to effectively inhibit harmful microorganisms in silage, leading to the decomposition of protein. In contrast, high concentration of LP can quickly create an acidic environment, significantly enhancing the inhibitory effect on harmful bacteria, thus inhibiting the decomposition of CP. The decrease in CP content in BL groups is due to BL’s strong protease activity, which promotes the breakdown of protein (Zhu et al., 2022), and as the increase of BL concentration, the CP content gradually decreases. In different treatment groups, NPN accounts for approximately 93.06 to 97.89% of the SCP content. The NPN in all experimental groups was lower than that in CON, while the TP content was higher than that in CON, indicating that both LP and BL can effectively prevent the degradation of protein into simple nitrogenous compounds such as ammonia in PS silage. According to the different degradation rates of various parts of feed in the rumen of ruminants, the CNCPS version 6.5 has categorized the protein components (Pan et al., 2016), which mainly include PA1, and the lower its value, the higher the content of true protein in the feed and the higher the feed value. In this experiment, the PA1 of all experimental groups was lower than that of the control group. PA2 refers to the soluble true protein in the feed, which degrades rapidly in rumen. PB1 refers to the insoluble true protein in the feed, and PB2 refers to the fiber-bound protein. PB1 and PB2 are the main protein that are digested in feed, and PB1 is mainly degraded in rumen, while PB2 is mainly absorbed in intestine (Sun and Yu, 2023). PC refers to the undigestible protein in rumen of feed. The higher the PC content, the lower the rumen degradation rate, and the poorer quality of feed protein. The PC content in ruminant feed should generally be under 10%. In this experiment, PB1 content in LP groups was significantly higher than that in the control group. PC content in LP7 group decreased by 19.95% compared to CON, indicating that high concentration of LP can change the composition of protein fractions in feed, increase the proportion of moderately degradable proteins, reduce the content of indigestible proteins in rumen, and improve the quality of feed protein. As the addition concentration increases, protease activity strengthens. PB1 content in BL groups gradually decreases, and PC content gradually increases, leading to a decline in feed protein quality. This suggests that low concentration of BL can improve nutritional value of feed protein. In summary, the higher the concentration of LP, the higher the protein quality, while the lower the addition of BL, the higher the nutritional value of protein, and LP7 performed better than BL5.

The high content of NDF and ADF will affect the feed intake and digestibility of animals (Grant and Ferraretto, 2018). In this experiment, the NDF and ADF contents of LP7 group were both significantly lower than those of CON, which is consistent with the research results of Zi et al. (2021). The addition of LP can promote the degradation of fiber in silage, and the HC content in LP groups decreased with the increase of LP concentration, indicating that LP can promote the degradation of HC. In this experiment, the ADF content of BL groups was significantly lower than that of CON. This is consistent with the research results of Mara et al. (2016), which found that *Bacillus licheniformis* has a strong ability to degrade fiber. Moreover, the HC content in BL groups was significantly higher than that in CON, possibly because BL primarily promotes the decomposition of cellulose. The NFC content in all experimental groups was higher than that in CON, possibly because LP and BL can promote the conversion of fiber carbohydrates into non-fiber carbohydrates in silage, which is consistent with the aforementioned results. CHO is primary energy source for ruminants. LP can increase the content of CHO, but as the LP addition increases, the activity of exogenous bacteria can lead to a gradual decrease in CHO content due to nutrient consumption. BL can inhibit the activity of microorganisms in silage, and quickly create an anaerobic environment, reducing nutrient consumption in feed. The higher the concentration, the faster it works and the higher the CHO content.

The nutritional value of CHO for ruminants mainly depends on the degradation degree of structural and non-structural carbohydrates in the rumen. CNCPS 6.5 divides the CHO components in feed into three parts: CA, CB and CC (carbohydrates that are difficult to utilize). Among them, CA further divided into CA1 (mainly including AA, PA and BA), CA2 (referring mainly to LA), CA3 (other organic acids), and CA4 (sugars). CB includes CB1 (starch), CB2 (soluble fiber) and CB3 (available NDF) (Gomaa et al., 2018; Van Amburgh et al., 2015). The changes in CA4 and CB1 fractions are mainly related to the regulation of the silage microflora by exogenous bacteria and the consumption of carbohydrates and degradation of macromolecular fiber substance by microbial activity. The increase in CB2 and decrease in CB3 content in all treatments indicate that LP and BL can alter the composition of carbohydrates in PS silage. The higher CC content in all treatment groups compared to CON may be due to the promotion of fiber degradation in silage by LP and BL, reducing the content of other types of fiber in silage carbohydrates, which leads to an increase in the relative content of CC.

The silage process is often accompanied by intense microbial activities, and the use of additives often has a great influence on the microbial community of silage (Wang et al., 2019). Through 16S rRNA high throughput sequencing technology, we can have a deeper understanding of how additives improve the quality of silage by affecting the microbial community. Goods refers to the coverage of sample library, and the higher the value of goods, the higher the reliability of results (Dong et al., 2020). Goods in all treatment groups was above 0.99, indicating that the sequencing data coverage was sufficient to reflect the silage microbial community. The Ace and Chao1 index represent species richness, with higher values indicating greater community richness. Numerous studies have shown that *Bacillus licheniformis* has strong antimicrobial activity, and its metabolic products can inhibit the growth of harmful bacteria such as *Clostridium perfringens* and *Staphylococcus aureus in vitro* (Musa et al., 2019; Shobharani et al., 2015). This is consistent with the Ace and Chao1 index results of this experiment, suggesting that *Bacillus licheniformis* may inhibit the growth of harmful bacteria through its fermentation products, reducing the abundance of bacterial colonies in silage. The Simpson and Shannon index are used in evaluating species diversity (Dong et al., 2020; Tian et al., 2021). As the concentration increases, Shannon index of LP groups continues to decrease, and Shannon index of LP7 is significantly lower than that of CON and LP5, indicating that LP can inhibit the proliferation of miscellaneous bacteria, reducing species richness in silage. Simpson index of BL groups is significantly higher than that of CON, indicating that BL can promote the enrichment of dominant bacteria in silage, increasing species evenness. The results of α-diversity suggest that LP and BL have different impacts on the microbial colony of PS silage. β-diversity reflects the differences in bacterial communities between samples, and its results also lead to the same conclusion. From the PCoA figure, we can see that there is an obvious separation between CON and experimental group. And with the increase of concentration, the LP and BL groups are also gradually separated, indicating that the addition of different additives has a different impact on the microbial community of silage, and this impact is related to the concentration of additive.

The application of additive can not only change the richness and diversity of bacteria but also change the composition and structure of the bacterial community. Reduced diversity of bacterial community could signal dominance by beneficial taxa (e.g., *Lactobacillus*), improving fermentation efficiency. Conversely, higher diversity might indicate incomplete suppression of undesirable microorganisms. At the phylum level, the main phyla in each treatment group were *Firmicutes*, which was consistent with the results of Chen et al. (2020) and Zi et al. (2021). *Firmicutes* mainly includes spore-producing, non-spore-producing and mycoplasma bacteria, which can degrade macromolecular substances, such as cellulose, starch, protein, etc. Some species in *Proteobacteria* can undergo deamination reactions to produce ammonia, hindering the decrease of pH (Silva et al., 2016). Nitrogen is one of the most important elements in the growth process of organisms. Study has shown that *Cyanobacteria* can use ammonia and simple nitrogen-containing compounds as nitrogen source to promote growth (Herrero et al., 2001). In this experiment, the relative abundance of *Cyanobacteria* in each experimental group was higher than that in CON, and the relative abundance of *Firmicutes* was lower than that in CON. This may be because LP and BL promoted the decomposition of proteins or other nitrogen-containing macromolecules into simple nitrogen-containing compounds in silage, which were further decomposed into ammonia under the action of *Proteobacteria*, providing adequate nitrogen source for *Cyanobacteria* and promoting its rapid proliferation, thereby leading to a decrease in the relative abundance of *Firmicutes*. In addition, LP and its metabolites can directly inhibit the growth of harmful bacteria in *Proteobacteria*, such as *Vibrio*, *Spirillum*, *Escherichia coli* and *Salmonella*. In this experiment, with the increase of LP concentration, the relative abundance of *Proteobacteria* gradually decreased. *Bacteroidetes* can utilize and degrade organism (Kirchman, 2002). The relative abundance of *Bacteroidetes*, *Acidobacteria*, *Planctomycetes*, *Patescibacteria*, *Chloroflexi* in all experimental groups is significantly decreased, indicating that the addition of LP and BL can effectively reduce the relative abundance of non-silage main functional bacteria in silage, thereby reducing the loss of nutrients during silage. In addition, the differences in microbial communities among treatment groups with varying concentrations may be due to differences in the metabolism of specific species. LP rapidly produces LA, favoring acid-tolerant microbial groups while inhibiting acid-sensitive ones (Evelaine et al., 2023). High-dose strains may accelerate the decrease in pH, leading to a sharp decline in diversity. BL might secrete antimicrobial peptides or enzymes (e.g., proteases), selectively inhibiting certain phyla (e.g., *Bacteroidetes*) while promoting others (Zhu et al., 2022). Strains with stronger antimicrobial activity may target specific competitors.

The dominant bacteria genus in each treatment group was *Lactobacillus*, which was consistent with the results of Shah et al. (2020). The dominant bacteria genus on the surface of PS silage was *Lactobacillus*. *Lactobacillus* is the main bacteria of silage fermentation, its fermentation product LA can quickly reduce the pH of silage. LA exhibits selectivity due to the differential acid tolerance among microorganisms. Pathogens like *Clostridium* lack acid resistance mechanisms, whereas LAB tolerate acidity via proton pumps (Evelaine et al., 2023). Moreover, LAB preferentially utilize soluble sugars, thereby restricting carbon source availability for pathogenic microorganisms such as yeasts and molds (Marbun et al., 2020). Consequently, low PH preferentially restricts competitive microbiota while allowing acid-tolerant beneficial species to dominate, thereby improving both the success rate and fermentation quality of silage. Compared to CON, the addition of LP and BL both altered the composition of microorganisms in silage, reducing the relative abundance of *Lactobacillus* and increasing the relative abundance of the *Leuconostoc*. This may be because LP competes with certain strains of the native *Lactobacillus* in PS silage, leading to an overall decrease in the relative abundance of *Lactobacillus* in LP groups. BL groups may be due to the bacteriostatic ability of BL and its metabolic products, which directly inhibit the activity of the native *Lactobacillus* in silage. Some studies report that *Leuconostoc* is a common species in silage, belonging to the heterofermentative LAB, and its metabolic pathway for glucose is 6-phosphogluconate pathway, with the end products being LA and ethanol (Zhu et al., 2022). *Lactococcus* is a facultative anaerobe and is also one of the common species in silage, and its primary glucose fermentation product is LA (Whitman, 2016). *Weissella* is one of the most common heterofermentative bacteria in silage, which can produce same amount AA as LA. With the accumulation of organic acids and the decrease of pH, its growth will be inhibited (Muck, 2013). From the results of this experiment, it can be seen that LP can increase the relative abundance of *Leuconostoc* and *Lactococcus*, and as the concentration increases, the relative abundance of *Lactobacillus* in silage increases, and the relative abundance of *Weissella* decreases, thereby increasing the content of LA and reducing the content of AA, improving the quality of silage. BL can also increase the relative abundance of *Leuconostoc* in silage and reduce the pH, and as the concentration increases, the relative abundance of *Weissella* decreases. In addition, the addition of LP and BL both reduced the relative abundance of genera such as *Sediminibacterium*, *Raoultella* and *Sphingomonas*, which are mainly found in soil and have strong degradation capabilities, possibly related to the metabolism of nutrients in feed. The decrease in their abundance indicates that LP and BL can reduce the activity of microorganisms in silage and decrease nutrient loss. Studies report that *Acinetobacter* contains a variety of pathogenic bacteria, the presence of which can lead to serious diseases of lungs, skin, nerves, etc. (Harding et al., 2018; Peleg et al., 2008). Compared with CON, the relative abundance of *Acinetobacter* in all treatment groups significantly decreased, indicating that LP and BL can inhibit the activity of pathogenic bacteria in silage, improve the safety of silage and provide healthy feed for animals.

## 5 Conclusion

In conclusion, LP and BL have significant effects on silage quality and nutritional value, and their application is worthy of promotion in practical production. The nutritional value of *Pennisetum sinese* in LP groups showed a dose-dependent effect, and adding 1 × 107 CFU/g LP have the best effect in silage. The best effect was achieved by adding 1 × 105 CFU/g BL in BL groups, and the effect of LP7 was better than that of BL5. And LP and BL can significantly change the structure of microbial community and reduce the relative abundance of harmful microorganisms in silage, and the change of microbial community structure is affected by the inoculants and concentration. These findings challenge the traditional view of silage additives as mere pH modifiers, positioning them instead as ecological engineers that steer microbiome assembly. Future research should explore LP-BL consortia to harness specific advantages during ensiling dynamics.

## Data Availability

The data and materials presented in this study are available on request from the corresponding authors.
